# Transcriptomic Characterization of Copper-Binding Proteins for Predicting Prognosis in Glioma

**DOI:** 10.3390/brainsci13101460

**Published:** 2023-10-14

**Authors:** Hao-Long Zeng, Huijun Li, Qing Yang, Chao-Xi Li

**Affiliations:** 1 Department of Laboratory Medicine, Tongji Hospital, Tongji Medical College, Huazhong University of Science and Technology, Wuhan 430030, China; lihuijun@tjh.tjmu.edu.cn; 2 Institute of Food Science and Engineering, Wuhan Polytechnic University, Wuhan 430023, China; qingyang@whu.edu.cn; 3 Department of Neurosurgery, Tongji Hospital, Tongji Medical College, Huazhong University of Science and Technology, Wuhan 430030, China; cxli@tjh.tjmu.edu.cn

**Keywords:** copper binding, glioma, prognosis, bioinformatics, risk model

## Abstract

Background: Copper and copper-binding proteins are key components of tumor progression as they play important roles in tumor invasion and migration, but their associations in gliomas remain unclear. Methods: Transcriptomic datasets of glioblastoma, low-grade glioma, and normal brain cortex were derived from the TCGA and GTEX databases. Differentially expressed genes (DEGs) of copper-binding proteins were screened and used to construct a prognostic model based on COX and LASSO regression, which was further validated by the CGGA datasets. The expressions of risk-model genes were selectively confirmed via anatomic feature-based expression analysis and immunohistochemistry. The risk score was stratified by age, gender, WHO grade, IDH1 mutation, MGMT promoter methylation, and 1p/19q codeletion status, and a nomogram was constructed and validated. Results: A total of 21 DEGs of copper-binding proteins were identified and a six-gene risk-score model was constructed, consisting of ANG, F5, IL1A, LOXL1, LOXL2, and STEAP3, which accurately predicted 1-, 3-, and 5-year overall survival rates, with the AUC values of 0.87, 0.88, and 0.82, respectively. The high-risk group had a significantly shorter OS (*p* < 0.0001) and was associated with old age, wild-type IDH1, a high WHO grade, an unmethylated MGMT promoter, and 1p/19q non-codeletion and had higher levels of immune cell infiltration, cancer-immunity suppressor, and immune checkpoint gene expression as well as a higher TMB. Conclusions: The model based on the genes of copper-binding proteins could contribute to prognosis prediction and provide potential targets against gliomas.

## 1. Introduction

Copper is an essential nutrient; however, tumors are especially dependent on the metal, as angiogenesis could be promoted [1]. In addition, copper is thought to be a key component of cancer metastasis because of its important role in invasion/migration processes [2]. Among primary brain tumors, gliomas are the most common type [3], and they consist of lower-grade gliomas (LGGs) (WHO grade II and III) and glioblastoma (GBM) (WHO grade VI), with a median OS time of 78.1, 37.6, and 14.4 months for WHO grades II, III, and IV, respectively [4]. Considerable evidence has shown that anticancer therapy targeting copper is worthy of in-depth study in glioma [5], including the inhibition of tumorigenesis with copper-specific chelators or with copper-containing ionophores [6]. Since most of the copper in the body is protein-bound, how these copper-binding proteins, such as extracellular lysyl oxidase (LOX), participate in tumorigenesis still needs to be clarified [7]. In addition, copper-binding proteins have also emerged as potential prognostic markers [8], which are still lacking in sufficient elucidation.

In this study, we performed a bioinformatic analysis of copper-binding proteins based on the Cancer Genome Atlas (TCGA) datasets and constructed a prognostic model and validated it with the Chinese Glioma Genome Atlas (CGGA) datasets. This risk-score model accurately predicted overall survival rates, with high sensitivities and specificities, and the high-risk group showed a significantly shorter OS, and higher levels of immune cell infiltration, cancer-immunity suppressor, and immune checkpoint gene expression, as well as a higher tumor mutational burden (TMB). This study could be helpful for the prognosis prediction and management of patients with gliomas.

## 2. Materials and Methods

### 2.1. Copper-Binding Protein Gene Sets

Copper-binding protein gene sets were obtained from five gene sets from the MSigDB (https://www.gsea-msigdb.org/gsea/msigdb, accessed on 4 April 2023): GOMF_COPPER_ION_BINDING, GOBP_COPPER_ION_IMPORT, GOBP_COPPER_ION_TRANSPORT, GOBP_COPPER_ION_TRANSMEMBRANE_TRANSPORT, and GOMF_COPPER_ION_TRANSMEMBRANE_TRANSPORTER_ACTIVITY. Additionally, several other copper-binding proteins were also added according to a previous report [9]. Finally, a total of 85 genes were confirmed after removing overlapping genes, and they are listed in Table S1.

### 2.2. Patient Datasets

The TCGA RNA-seq datasets were downloaded from UCSC Xena (https://xenabrowser.net/datapages/, accessed on 4 April 2023), including 506 LGG samples and 154 GBM samples. The clinical features of the TCGA GBM and LGG patients were acquired from the UCSC Xena platform and from previous reports [10]. The Chinese Glioma Genome Atlas (CGGA) mRNA expression data (mRNAseq_325) for 325 glioma patients and their clinicopathological features were collected (http://www.cgga.org.cn, accessed on 4 April 2023) [11]. The normal cerebral cortex transcriptome datasets were acquired from the GTEX project, including 263 normal cerebral cortex samples. Both of the two types of data, counts and FPKM, were downloaded. The counts data were used for differential analysis using the DESeq2 R package, while the FPKM data were used for risk score construction.

### 2.3. Differential Analysis

The differential analysis was performed using the DESeq2 R package, between the TCGA GBM, LGG, and GTEX samples, respectively, using the datasets of the HTSeq-Counts. Differentially expressed genes (DEGs) were identified by a false discovery rate (FDR) <0.05 and absolute foldchange >1.5. With respect to copper-binding proteins, their related DEGs identified in each of the three comparisons (GBM vs. GTEX, LGG vs. GTEX, or GBM vs. LGG) were included for further analysis.

### 2.4. Constructing and Validating the Risk-Score System

The HTSeq-FPKM datasets were used for risk score construction. Univariate Cox regression was first performed for the copper-binding protein-related DEGs. Then, least absolute shrinkage and selection operator (LASSO) regression was performed using the glmnet R package. Multivariate Cox regression was then performed for the identified genes, and the risk score was calculated as below:

$$Risk\;score=\sum_{i=1}^{n}{expr}_{\;genei}\times {coefficient\;}_{genei}$$

The TCGA and CGGA datasets were used as training and validating sets, respectively. The receiver operating characteristic (ROC) curve was plotted using timeROC R package. According to the median risk score, patients were divided into high- or low-risk groups, and their overall survival (OS) rates were evaluated via Kaplan–Meier survival analysis. Only samples with survival data were used in the analysis.

### 2.5. Development and Evaluation of the Nomogram

To evaluate the predictive values of the risk score system, univariate and multivariate Cox regression analyses were performed using the following variables: age, gender, WHO grade, isocitrate dehydrogenase 1 (IDH1) mutation status, 1p19q codeletion status, and O^6^-methylguanine-DNA methyl-transferase (MGMT) promoter methylation status. All parameters were used to construct a nomogram using the rms R package. Concordance index (C-index), calibration, decision curve analysis (DCA), and ROC analyses were used to evaluate the nomogram.

### 2.6. GSEA, Immune Cell Infiltration, and Immune Subtypes

The GSEA was performed using the clusterProfiler package [12]. The ssGSEA method of the GSVA R package was used to analyze the immune infiltrates [13]. The immune subtypes were identified using the R ImmuneSubtypeClassifier [14], with six immune subtypes established according to previous report [14], including C1: Wound Healing; C2: IFN- γ Dominant; C3: Inflammatory; C4: Lymphocyte Depleted; C5: Immunologically Quiet; C6: TGF- β Dominant.

### 2.7. Mutation Landscape and Tumor Mutation Burden (TMB)

The gene mutation was analyzed via maftools R package [15]. The R packages TCGAmutations and maftools were used to perform the TMB analysis.

### 2.8. Anatomic Transcriptional Expression of Prognostic Genes

The anatomic transcriptional expression was analyzed by using the Ivy Glioblastoma Atlas Project (Ivy GAP) (http://glioblastoma.alleninstitute.org/, accessed on 4 April 2023) [16], which summarized the transcriptomes of seven anatomic features, including leading edge (LE), infiltrating tumor (IT), cellular tumor (CT), pseudopalisading cells around necrosis (PAN), perinecrotic zone (PNZ), microvascular proliferation (MVP), and hyperplastic blood vessels (HBVs). The LE is the outermost boundary of the tumor (peritumoral zone).

### 2.9. Immunohistochemistry

The immunohistochemical staining of STEAP3 and LOXL1 were performed in glioma patients who received surgical tumor resection in Tongji Hospital (Wuhan, China). The peritumoral normal brain tissue was resected in very small amounts from the brain area that surrounded the tumor and only from non-eloquent cerebral areas. Sections of 3 μm were subjected to the immunohistochemical staining by using the monoclonal antibody anti-STEAP3 (EPR9812, Abcam, Cambridge, UK) and anti-LOXL1 (ab238152, Abcam, Cambridge, UK). Approval was obtained from the Ethics Committee of Tongji Hospital, Tongji Medical College, Huazhong University of Science and Technology in Wuhan, China. All the procedures involving human samples conformed to the principles outlined in the Declaration of Helsinki. 

### 2.10. Statistical Analysis

All statistical analyses were conducted using R software (version 4.3.0). Quantitative data are presented as the mean ± standard error (SEM) or standard deviation (SD). The Wilcoxon test was applied to compare the statistical differences between the two groups. Statistical significance was defined as *p* < 0.05.

## 3. Results

### 3.1. Transcriptomic Expressions of Copper-Binding Proteins

**Figure 1:**
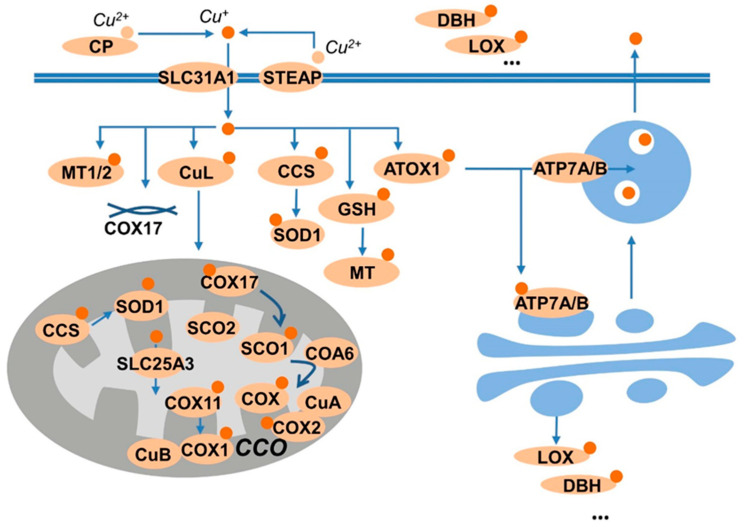
Summary of copper-binding proteins. Copper is carried by ceruloplasmin (CP), and uptake by the transporter SLA31A1 (CTR1) or SLC11A2 (DMT1). The transporters prefer Cu^+^ as substrate; thus, the reductase, like STEAP provide the reduced species for uptake. The Cu^+^ is then sequestered by GSH, or stored in metallothioneins (MTs), or shuttled to the cellular targets by the chaperones CCS to superoxide dismutase 1 (SOD1) and by antioxidant protein 1 (Atox1) to ATP7A/B. ATP7A/B transport copper into the trans-Golgi network for subsequent incorporation into copper-dependent enzymes such as CP, dopamine-β-monoxygenase (DBH), or lysyl oxidase (LOX). Moreover, the copper taken up by cells also binds to copper ligands (CuLs) and transports to the mitochondria, in which, copper chaperone for cytochrome C oxidase 17 (COX17) supplies two pathways: delivering copper to COX11 and the synthesis of cytochrome oxidase1 (SCO1).

A total of 85 proteins were confirmed to possess copper-binding capability (Table S1). These proteins mainly consist of copper-dependent enzymes, including cytochrome c oxidase (CCO), superoxide dismutase 1 (SOD1), oxygenase/oxidase like tyrosinase, lysyl oxidase (LOX), dopamine β- hydroxylase (DBH), copper amine oxidases, etc. In addition, copper transporters, including copper transporter 1 (SLC31A1/CTR1) and copper-transporting P-type ATPase ahpha and beta (ATP7A and ATP7B), and copper chaperones, including copper chaperone for superoxide dismutase 1 (CCS) and antioxidant-1 (Atox-1), and other copper-binding proteins like metallothionein, etc., were also included since the bioavailability of intracellular copper is tightly controlled by these factors. These copper-binding proteins were mostly involved in copper homeostasis, as shown in Figure 1.

**Figure 2:**
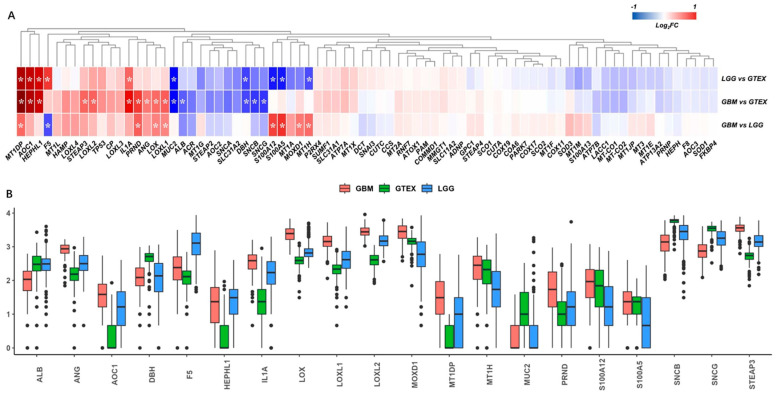
Differential analysis of copper-binding protein gene sets. (**A**) The copper-binding protein gene sets were extracted from the TCGA-GBM, TCGA-LGG, and GTEX normal-brain cortex datasets, and a total of 78 genes matched. *, *p* < 0.05 and fold-change >1.5 or <−1.5. (**B**) The differential analysis identified 10 differentially expressed genes (DEGs) in the comparisons of LGG vs. GTEX, 15 DEGs in GBM vs. GTEX, and 9 DEGs in GBM vs. LGG, respectively, according to the criteria for DEGs. Collectively, a total of 20 DEGs were identified and are shown as a boxplot to compare the expressions across the TCGA-GBM, TCGA-LGG, and GTEX samples.

The gene expressions of the copper-binding proteins were extracted from the TCGA-GBM, TCGA-LGG, and GTEX normal-brain cortex datasets, and a total of 78 genes matched. The PCA and heatmap showed distinct profiles among the three datasets (Figure S1). After differential analysis, we identified 10 DEGs in the comparisons of LGG vs. GTEX, 15 DEGs in GBM vs. GTEX, and 9 DEGs in GBM vs. LGG, respectively (Figure 2A). Collectively, a total of 20 DEGs were identified, as shown in the boxplot (Figure 2B).

### 3.2. Construction and Validation of the Prognostic Model

**Figure 3:**
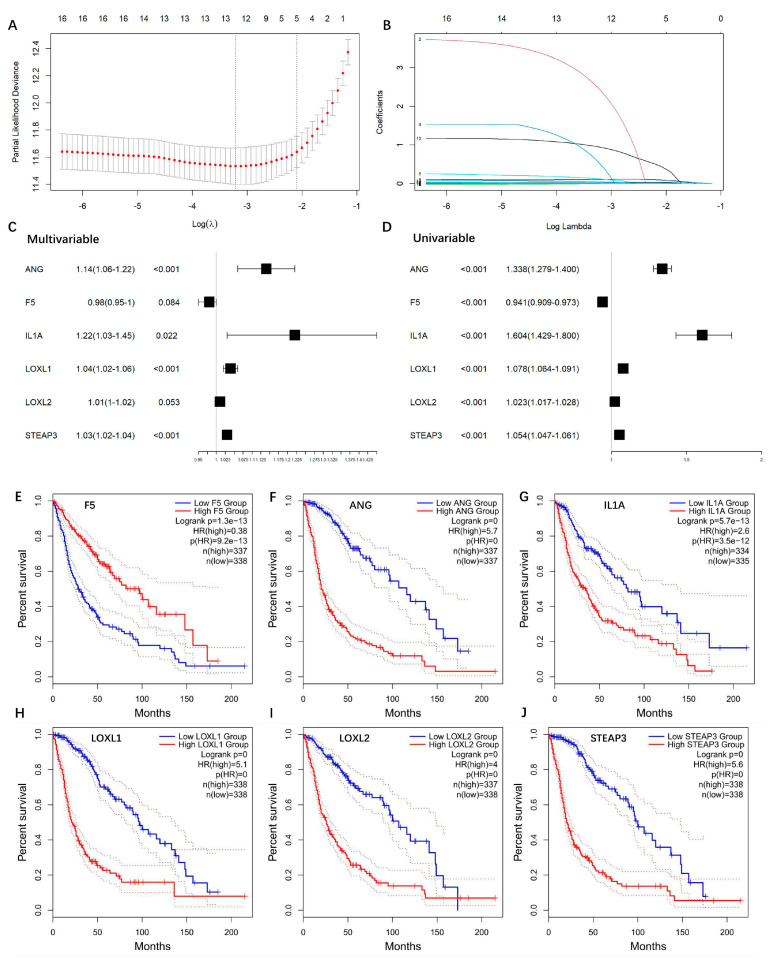
Construction of copper-binding protein-related prognostic model. (**A**) LASSO regression was used to calculate regression coefficients, and 12 genes were returned as the most valuable predictive genes, with λ of −3.2 at the minimum partial likelihood deviation. (**B**) Path diagram of LASSO regression coefficients. The colored lines are the paths of regression coefficients shrinking toward zero. (**C**) The AIC method of multivariate Cox regression analysis was performed to further optimize the model, and a total of six genes (ANG, F5, IL1A, LOXL1, LOXL2, STEAP3) were finally included. (**D**) Forest plots of the univariate Cox regression analysis. (**E**–**J**) Univariate Kaplan–Meier survival plots of ANG, F5, IL1A, LOXL1, LOXL2, STEAP3, respectively.

To investigate the relationship between the gene expressions and overall survival time, we firstly performed univariate Cox regression for each of the 20 DEGs, respectively. Sixteen genes showed significance (*p* < 0.05) and as candidate risk factors related to OS (Table S2). Then, LASSO regression was performed to refine these genes by calculating regression coefficients (Figure 3A,B), and 12 genes were returned. Multivariate Cox regression analysis was performed to further optimize the model, and a total of six genes (ANG, F5, IL1A, LOXL1, LOXL2, STEAP3) were finally included. The multivariate and univariate Cox regression results of the six genes are summarized in Figure 3C,D. Among these six genes, ANG, IL1A, LOXL1, LOXL2, and STEAP3 played risk roles in the survival of glioma patients (HR > 1), while F5 was a potential protective factor (HR < 1). These genes were further confirmed via univariate Kaplan–Meier survival analysis (Figure 3E–J).

**Figure 4:**
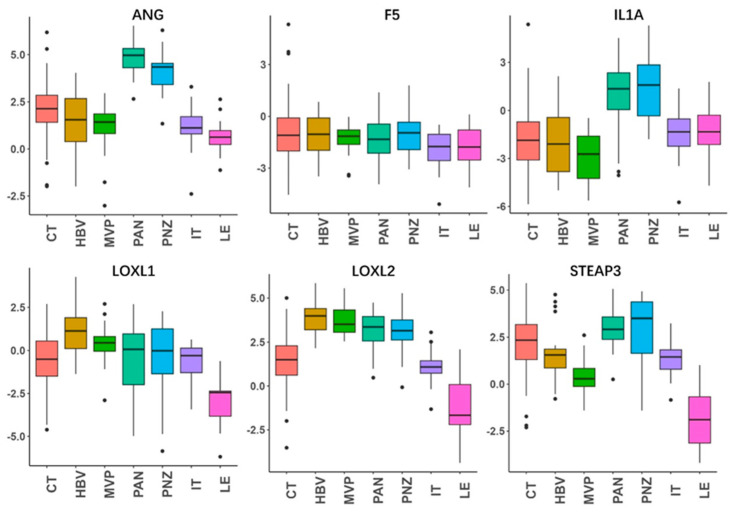
Anatomic expressions of the genes in the risk-score model. By using the anatomic transcriptional atlas from Ivy GAP, the relative anatomic expressions of ANG, F5, IL1A, LOXL1, LOXL2, and STEAP3 were assessed. Leading edge (LE), infiltrating tumor (IT), cellular tumor (CT), pseudopalisading cells around necrosis (PAN), perinecrotic zone (PNZ), microvascular proliferation (MVP), and hyperplastic blood vessels (HBVs). The LE is the outermost boundary of the tumor (peritumoral zone), where the ratio of tumor to normal cells is about 1–3/100, and the layers of the cortex are often observed.

**Figure 5:**
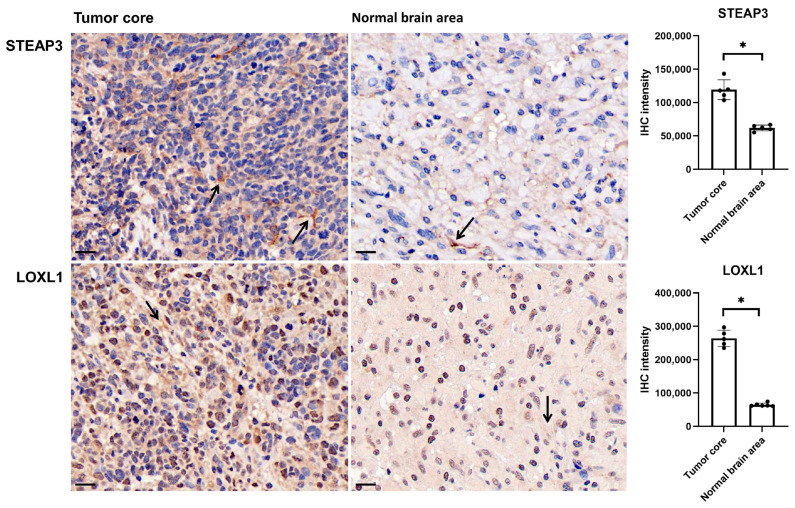
STEAP3 and LOXL1 expression in tumor core and normal brain area in glioma. Sections of 3 um were subjected to the immunohistochemical staining (arrow) by using the monoclonal antibody anti-STEAP3 and anti-LOXL1. Scale bar: 20 um, * *p* < 0.05.

We further analyzed the anatomic transcriptional expressions of the six genes by using the Ivy GAP [16] (Figure 4). As expected, the risk factors ANG and IL1A showed the highest expressions in the necrosis-related area (PAN and PNZ) within the tumor zone. The other risk factors LOXL1, LOXL2, and STEAP3 were highly expressed in almost all tumor zones (CT, HBVs, MVP, PAN, PNZ, IT) and showed the lowest expression in the peritumor zone (LE), while the protective factor F5 showed no obvious change between the tumor and peritumor zone. To further verify the expressions of the prognostic genes, immunohistochemistry was also performed to evaluate the protein expressions of STEAP3 and LOXL1 (Figure 5). And as expected, STEAP3 and LOXL1 were significantly increased in the tumor core compared to the normal brain area.

**Figure 6:**
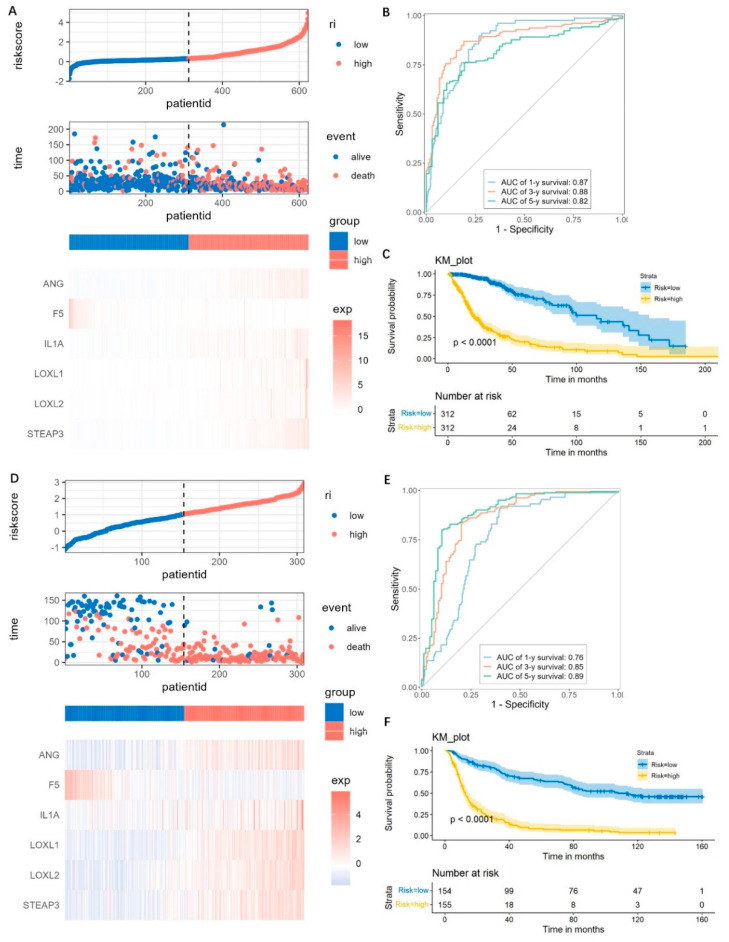
Prediction performance assessment and validation of the prognostic model. (**A**–**C**) Risk score distributions, expression heatmap, ROC curves, and Kaplan–Meier survival plot for predicting the 1-, 3-, and 5- year OS times based on the prognostic genes in the TCGA cohorts. (**D**–**F**) The prediction performance validation by using the CGGA cohorts.

Based on the six genes, we calculated the risk score for each glioma patient, and the risk score that is higher or lower than the median was defined as either high- or low-risk, respectively. The high-risk patients demonstrated increased risks of death and poor survival outcomes (Figure 6A). The gene expressions showed obviously higher levels of ANG, IL1A, LOXL1, LOXL2, STEAP3, and lower expressions of F5, in the high-risk group compared to the low-risk group (Figure 6A). The time-dependent ROC was analyzed, and the area under curve (AUC) values of 1 year reached 0.87, 3 year reached 0.88, and 5 year reached 0.82 (Figure 6B). And the high-risk group had a significantly shorter OS time than the low-risk group (*p* < 0.0001) (Figure 6C). 

For validations, the CGGA dataset was used and also divided into high-risk and low-risk groups as mentioned above. The distributions of risk score, survival time, and the gene expressions were similar with those observed in the TCGA datasets (Figure 6D). The AUC of the 1-, 3-, and 5-year prognoses were 0.76, 0.85, and 0.89, respectively (Figure 6E). Patients in the high-risk group have a significantly worse prognosis, which was similar with the TCGA dataset (Figure 6F). These results suggested that the risk score model was robust.

### 3.3. Clinically Stratified Analysis

**Figure 7:**
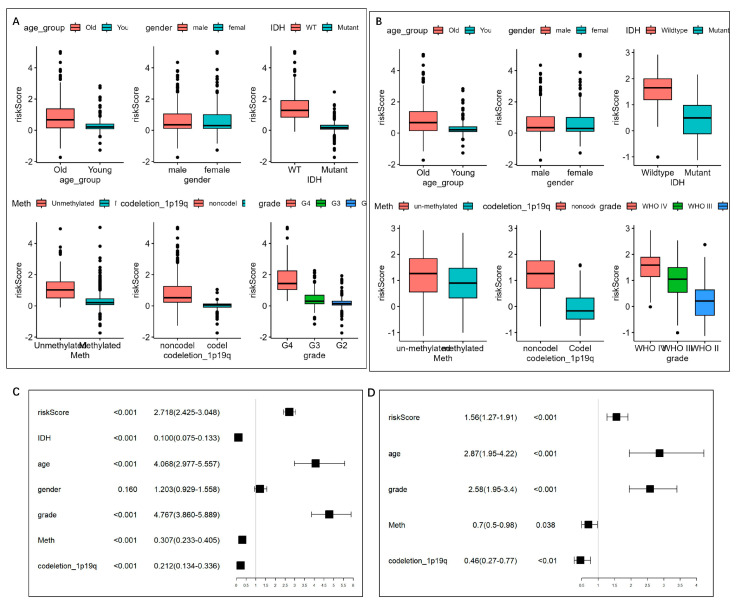
Risk score distribution and univariate/multivariate Cox regression analysis based on the clinical features. (**A**,**B**) Distribution of the risk scores in (**A**) TCGA and (**B**) CGGA cohorts. The risk score was grouped by age group, gender, IDH mutation status, MGMT promoter status, and 1p19q codeletion status. (**C**) Univariate Cox regression analysis of age, gender, WHO grade, IDH1 mutation status, MGMT promoter status, 1p/19q status, and risk level in TCGA datasets. (**D**) Multivariate Cox regression analysis on the single indicator with Cox *p* < 0.05, while variance inflation factor (VIF) < 2, so that five indicators, including age, WHO grade, MGMT promoter status, 1p/19q codeletion status, and risk score, were left.

As shown in Figure 7A,B, it could be observed that, consistently in the TCGA and CGGA datasets, patients with higher risk scores tend to be old (>40 years), and with a higher WHO grade, unmethylated MGMT promoter, non-codeletion of 1p/19q, and wild-type IDH1 (*p* < 0.05). However, no difference was found in the risk scores between genders. 

### 3.4. Construction and Validation of the Nomogram

**Figure 8:**
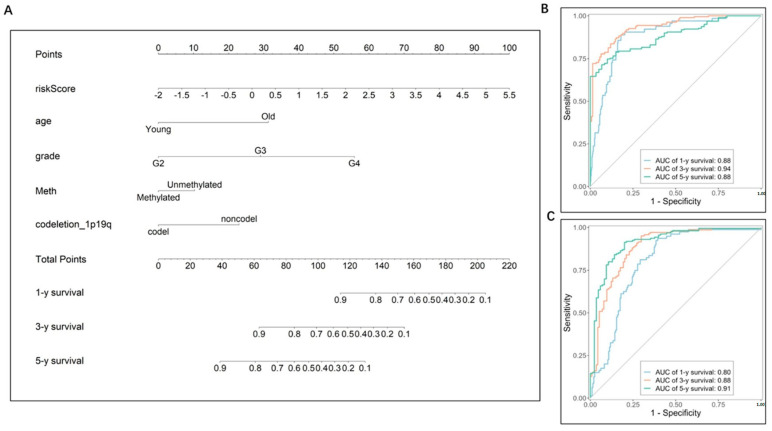
Prognostic nomogram for the 1-, 3-, and 5-year OS times. (**A**) Nomogram was constructed by integrating independent risk factors. The C-index of the nomogram model was 0.852 (95% CI = 0.839 − 0.865). (**B**) ROC curves of the nomogram in the TCGA cohort. (**C**) ROC curves of the nomogram in the CGGA cohort.

To evaluate the independence of the risk score, we analyzed the abovementioned potential indicators using univariate Cox regression analysis (Figure 7C). Multivariate Cox regression analysis was further performed on the single indicator with Cox *p* < 0.05, while the indicators with variance inflation factor (VIF) >2 was excluded to prevent multicollinearity, thus five indicators, including age, WHO grade, MGMT promoter status, 1p/19q codeletion status, and risk score, were left (Figure 7D) and were integrated into the nomogram model (Figure 8A). The C-index was 0.852 (95% CI = 0.839 − 0.865). According to the nomogram, the ROC curve was analyzed. In the TCGA set, the AUC reached 0.88 at 1-year, 0.94 at 3-year, and 0.88 at 5-year OS rates, respectively (Figure 8B). The calibration analysis showed good fittings between the observed value and optimized value of 1-, 3-, and 5-year OS rates (Figure S2A). And the DCA demonstrated that the nomogram performed well in predicting the OS rates (Figure S2B).

For a validation of the nomogram, the CGGA datasets were analyzed. The AUCs for the 1-, 3-, and 5-year OS rates were 0.80, 0.88, and 0.91, respectively (Figure 8C). The calibration curves showed good agreements between the predicted OS rates and the probabilities of the OS rates (Figure S2C), and the DCA demonstrated good clinical utility of the nomogram (Figure S2D). These results suggest good predictive ability of the nomogram. 

### 3.5. GSEA

To explore the difference between the high- and low-risk groups, GSEA was performed. Genes highly expressed in the high-risk group were significantly enriched in GO terms like ‘Adaptive immune response’, ‘Embryonic skeletal system morphogenesis’, ’development’, ‘Anterior/posterior pattern specification’, ‘Regionalization’ (Figure S3A), and KEGG pathways like ‘Immune network for IgA production’, ‘Hematopoietic cell lineage’, and ‘Cytokine-cytokine receptor interaction’ (Figure S3B). These results suggest potential roles for copper-binding proteins in the immune responses of gliomas.

### 3.6. High Risk Score Demonstrates an Immunosuppressive Feature

**Figure 9:**
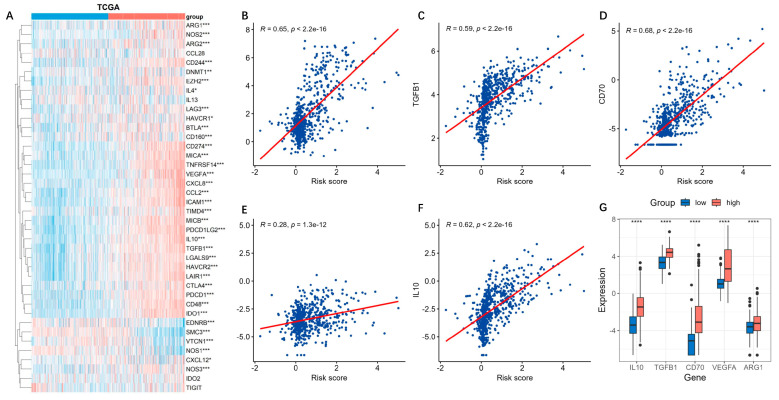
Expressions of inhibitory genes in cancer-immunity cycle based on the risk model. The cancer-immune cycle is a framework for how the immune system recognizes and kills cancer cells. (**A**) The expressions of the inhibitory genes in cancer-immunity cycle based on the risk model were analyzed by using the TCGA datasets. ***, *p* < 0.001; **, *p* < 0.01; *, *p* < 0.05. (**B**–**F**) The secreted immunosuppressive factors TGFB1, VEGFA, ARG1, IL10, and CD70 were positively correlated with the risk score. (**G**) Boxplot of the five immunosuppressive factors in the high-risk groups. ****, *p* < 0.05.

The cancer-immunity cycle is about how the immune system recognizes and kills cancer cells [17]. We explored the expressions of genes that inhibited this cycle [18]. We found that most of the genes were highly expressed in the high-risk group (Figure 9A). TGFB1, VEGFA, ARG1, and IL10 are secreted immunosuppressive factors in glioma, while CD70 is a glioma cell-surface immunosuppressive factor. These genes all seemed positively correlated with the risk score (Figure 9B–F), and were all obviously overexpressed in the high-risk groups (Figure 9G).

**Figure 10:**
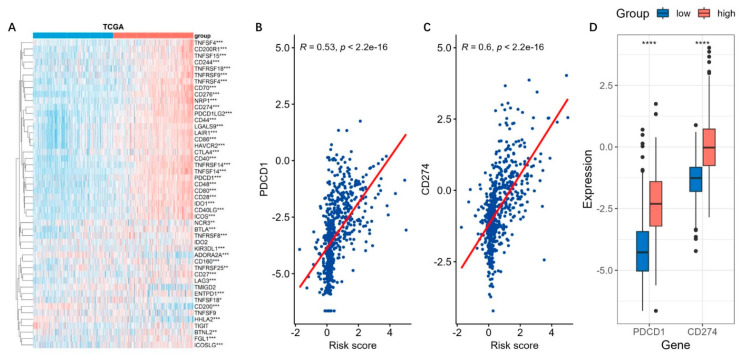
Immune checkpoint gene expressions based on risk model. (**A**) The expressions of the immune checkpoint genes based on the risk model were analyzed by using the TCGA datasets. ***, *p* < 0.001; **, *p* < 0.01; *, *p* < 0.05. (**B**,**C**) The immune checkpoint genes PDCD1 (PD-1) and CD274 (PD-L1) were positively correlated with the risk-score. (**D**) They were markedly higher in the high-risk groups. ****, *p* < 0.05.

Immune checkpoints suppress the immune system’s ability to clear tumors [19]. Similarly with findings above, most of the genes related to the immune checkpoints were up-regulated in the high-risk groups (Figure 10A). Among them, PDCD1 (PD-1) and CD274 (PD-L1), which play critical roles in tumor immunosuppression and immune therapy, were obviously positively correlated with the risk score (Figure 10B,C). Their expressions in the high-risk group were markedly higher than those in the low-risk group (Figure 10D). 

### 3.7. Immune Infiltration Analysis and Mutation Landscape

**Figure 11:**
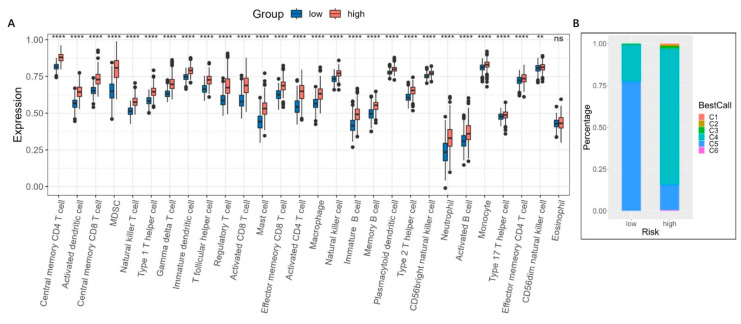
Immune infiltration and subtype analysis based on the risk model. (**A**) Comparison of the immunosuppressive cell levels in high- and low-risk groups. ****, *p* < 0.001; **, *p* < 0.01; ns, no significance. (**B**) Distribution of the immune subtypes using the ImmuneSubtypeClassifier R package. C1 (Wound healing): High angiogenic gene expression, proliferation rate, and Th2 cells; C2 (IFN-γ dominant): the highest M1/M2 macrophage polarization level with a strong CD8 signal; C3 (inflammatory): increased expression of Th17 and Th1 genes, low-to-moderate tumor cell proliferation; C4 (lymphocyte depleted): a more prominent macrophage signature, with Th1 suppressed and a high M2 response; C5 (immunologically quiet): the lowest lymphocyte count and the highest macrophage responses, dominated by M2 macrophages; C6 (TGF- β dominant): the highest TGF-β signature and a high lymphocytic infiltrate with an even distribution of type I and type II T cells.

We next compared the immune infiltration levels between the high- and low-risk groups. As shown in Figure 11A, higher levels of immune cell infiltration, including MDSCs, CD8+ T cells, regulatory T cells, neutrophils, macrophages, and NK cells could be observed in the high-risk group. To further explore the immune characteristics, the ImmuneSubtypeClassifier R package was used to classify different immune subtypes [14]. We found that both in the high- and low-risk groups, the main subtypes were C4 (lymphocyte depleted) and C5 (immunologically quiet), but many more C4 subtypes could be found in the high-risk group compared to the low-risk group (Figure 11B). The C4 subtype is characterized by a more prominent macrophage signature, with Th1 suppressed and a high M2 response, which has a worse prognosis than the C5 immune subtype in tumors [14]. This was consistent with the prognosis of high- and low-risk glioma patients. 

**Figure 12:**
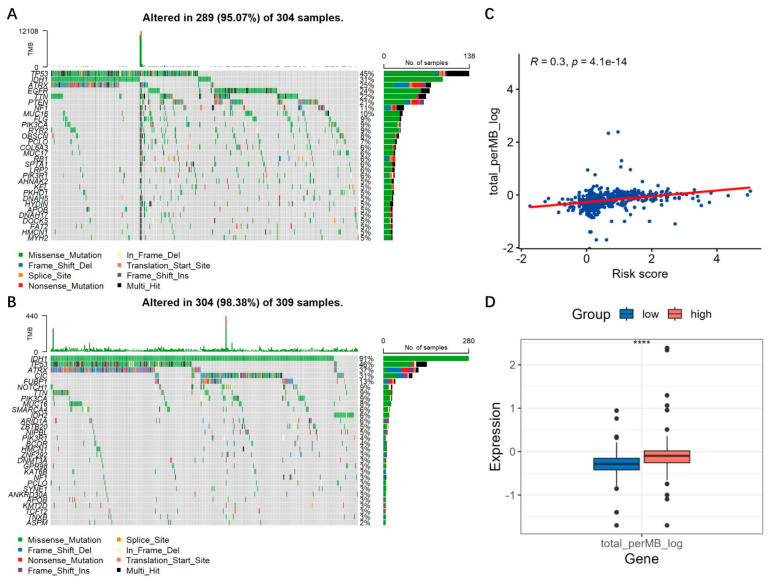
Mutation landscape and tumor mutation burden based on the risk model. (**A**,**B**) Mutation landscape of the high- and low-risk groups. (**C**) Correlation between TMB and risk scores. (**D**) Boxplot of TMB in the high- and low-risk groups. ****, *p* < 0.05.

We also analyzed the mutation landscape. In the low-risk group, the mutation incidence was 98.38% and the most significantly mutated gene was IDH1 (Figure 12B), while in the high-risk group, the mutation incidence was 95.07% and the most significantly mutated gene was TP53 (Figure 12A). It was reported that IDH1-mutant astrocytomas showed increased TP53 mutations [20]. Since TP53 remains one of the most common mutations in both IDH1 mutant and wild-type gliomas, we believe that high IDH1 mutations are not necessarily always accompanied by high TP53 mutations. Between risk score and TMB, TMB was positively correlated with risk score (R = 0.3, *p* < 0.0001) (Figure 12C) and was significantly higher in the high-risk group (*p* < 0.001) (Figure 12D). 

## 4. Discussions

Emerging evidence has confirmed that copper is a dynamic signaling metal and metalloallosteric regulator, linking to diverse cellular processes, including mitochondrial respiration, antioxidant defense, redox signaling, kinase signaling, autophagy, and protein quality control, etc. [21]. Cancer patients have aberrantly and intratumorally elevated systemic copper levels, which promote tumorigenesis, angiogenesis, tumor metastasis, and the recurrence of diverse human cancers [6]. In glioma, the changed copper levels in tumor tissues compared to adjacent tissue have been reported in several previous studies [22,23]. Copper is thought as a key component of cancer metastasis because of the important role in invasion/migration processes, and the abnormalities that induced intracellular copper accumulation are involved in tumor progression [2,8]. 

Copper ions in the body are mostly protein-bound, and specific transport systems existed to ‘chaperone’ the metal to targets to avoid the toxicity of free copper [24]; therefore, it is important to investigate what copper-binding proteins participate in cancer development and progression. In this study, we gathered all known copper-binding proteins and revealed their putative involvement in glioma, especially the predictive values for prognosis, by using the available database resources of RNA transcript levels. We confirmed a total of 85 genes with copper-binding capability by using the MSigDB along with manual curation. These genes mainly consist of copper-dependent enzymes, copper transporters, metallothionein, and other copper-containing factors. Based on these copper-binding proteins gene sets, we conducted a bioinformatic analysis using TCGA datasets, and screened six genes with prognostic value via LASSO and Cox regression analysis: F5, ANG, IL1A, LOXL1, LOXL2, and STEAP3, and constructed a prognostic model, which were further validated using the CGGA cohort.

Among the six genes of the risk model, F5, coagulation factor V, is a circulating procofactor in the blood coagulation cascade. It emerges as an interesting immunological biomarker with potential therapeutic relevance for the cancer–inflammation–thrombosis circuit [25]. ANG, angiogenin, is a multifunctional secreted ribonuclease that is upregulated in human glioblastoma that promotes glioblastoma progression by enhancing invasion, vascular association, proliferation, and survival [26]. IL1A, interleukin 1α, is a ubiquitous and pivotal pro-inflammatory cytokine, which modulates the glioma microenvironment and increases tumor invasion, migration, and angiogenesis [27]. LOXL1 and LOXL2 belong to the lysyl oxidase family. Lysyl oxidase is a secreted copper-dependent monoamine oxidase that catalyzes the covalent crosslinking of collagen and elastin via oxidation in the tumor microenvironment. Lysyl oxidase is closely related to the progression of glioma [28]. STEAP3 is a metalloreductase that belongs to the six-transmembrane epithelial antigen of prostate (STEAP) family. It is confirmed to be involved in numerous biological processes, including molecular trafficking, cell proliferation, and apoptosis. It promotes tumorigenesis and development mainly via metal ion reduction and cell proliferation [29]. Overall, our risk model consisted of a cytokine: IL1A, and two coagulation/angiogenesis genes: F5 and ANG, and three copper-dependent enzymes: LOXL1, LOXL2, and STEAP3. This combination incorporates immune and metabolic factors, not only accurately predicting the prognosis, but also distinguishing different glioma molecular subtypes, including IDH1 mutation, MGMT promoter methylation, and 1p/19q co-deletion.

As is well known, cancer metabolism plays a crucial role in sustaining tumorigenesis and survival, and is also involved in antitumor immune response [30]. In the high-risk group, biological processes or pathways related to immune response were enriched as expected. Previous studies have demonstrated the immunosuppressive nature of glioma, which regulates antitumor immune responses [31], because glioma cell expression increased levels of immunosuppressive factors, which limits the presentation of antigens. In the cancer-immunity cycle [17], we found genes that inhibit the cycle, including the glioma-secreted and cell-surface immunosuppressive factors, TGFB1, VEGFA, ARG1, IL10, and CD70, they were highly expressed in the high-risk group and were positively correlated with the risk score. In addition, immune checkpoints suppress the immune system’s ability to clear tumors [19], and most genes of the immune checkpoints were up-regulated in the high-risk groups, including PD1 and PD-L1. In theory, gliomas should be ideal candidates for immunotherapy since immune cells can cross the blood–brain barrier and selectively kill cancer cells while sparing normal brain cells; however, most glioma immunotherapy trials have shown only modest benefits, which are mostly due to this special immunosuppressive tumor microenvironment that has been deeply involved in the progression of glioma [32]. Furthermore, as expected, higher levels of immune cell infiltration in gliomas can be observed in the high-risk group, including myeloid-derived suppressor cells (MDSCs), neutrophils, macrophages, and NK cells, and the immune subtype analysis [14] showed, both in the high- and low-risk groups, that the main subtypes were C4 (lymphocyte depleted) and C5 (immunologically quiet), but with many more C4 subtypes in the high-risk group, suggesting a more prominent macrophage signature with Th1 suppressed and a high M2 response, as well as a worse prognosis in gliomas [14], and especially in the high-risk group.

According to the risk model, we also analyzed the tumor mutation burden, as tumors with an increased TMB are correlated with better prognosis after immunotherapy [33]. For gliomas, especially for GBM, one of the major obstacles in immunotherapy is the low TMB. Our data suggested a positive correlation between risk scores and TMB in gliomas, which indicate that the patient with a higher risk score may respond better. 

Our study comprehensively explored the relationship of the gene expression of copper-binding proteins with the glioma prognosis, clinicopathological features, and immune characteristics. However, it should be noted that the study still has some limitations. Firstly, some newly discovered copper-binding proteins may still be missed in our study. More importantly, although we validated our results using independent datasets, the findings and the mechanism still require thoroughly experimental validation and exploration in future studies.

In conclusion, based on the comprehensive analyses of copper-binding proteins’ gene sets in TCGA, CGGA, and GTEX datasets, we constructed a six-gene prognostic model in glioma. Using this model, we can well predict the prognosis and the possible immunotherapy response of glioma patients, which provides a new direction for future research on the management of glioma. 

## Data Availability

Data sharing is not applicable to this article as no new data were created or analyzed in this study.

## Supplementary Materials

The following supporting information can be downloaded at: https://www.mdpi.com/article/10.3390/brainsci13101460/s1, Figure S1: PCA and expression heatmap of transcriptomic expressions of the copper-binding proteins; Figure S2: Assessment of the nomogram; Figure S3: Functional enrichment analysis of the risk model; Table S1: List of copper-binding-related proteins; TableS2: Copper-binding protein-related deferentially expressed genes and their relationship with OS, and their coefficients in univariate Cox regression.

## Abbreviations

TCGA	The Cancer Genome Atlas
CGGA	Chinese Glioma Genome Atlas
GTEX	Genotype Tissue Expression Project
GBM	Glioblastoma
LGG	Low-grade glioma
MSigDB	The Molecular Signatures Database
LASSO	Least absolute shrinkage and selection operator
OS	Overall survival
GSEA	Gene set enrichment analysis
MGMT	O^6^-Methylguanine-DNA methyl-transferase
IDH1	Isocitrate dehydrogenase 1
TMB	Tumor mutation burden
DCA	Decision curve analysis
ANG	Angiogenin
F5	Coagulation factor V
IL1A	Interleukin 1 alpha
LOXL1	Lysyl oxidase-like 1
LOXL2	Lysyl oxidase-like 2
STEAP	Six-transmembrane epithelial antigen of prostate
