# Supplementary material for: Transcriptomic Characterization of Copper-Binding Proteins for Predicting Prognosis in Glioma
Source: Brain Sci. 2023 Oct 14;13(10):1460. doi: 10.3390/brainsci13101460 (PMC10605646; doi:10.3390/brainsci13101460)
Supplement: Supplementary file 1 [file brainsci-13-01460-s001.zip › brainsci-2581645-supplementary.pdf]

Supplementary tables: Table S1, Table S2

Table S1. List of copper binding-related proteins	
Symbol	Name
ACR	acrosin
ADNP	activity dependent neuroprotector ho
ALB	albumin
ANG	angiogenin
AOC1	amine oxidase copper containing 1
AOC2	amine oxidase copper containing 2
AOC3	amine oxidase copper containing 3
APOA4	apolipoprotein A4
ATOX1	antioxidant 1 copper chaperone
ATP13A2	ATPase cation transporting 13A2
ATP7A	ATPase copper transporting alpha
ATP7B	ATPase copper transporting beta
CCS	copper chaperone for superoxide dismutase
COA6	cytochrome c oxidase assembly factor
COMMD1	copper metabolism domain containing 1
COX11	cytochrome c oxidase copper chaperone COX11
COX17	cytochrome c oxidase copper chaperone COX17
COX19	cytochrome c oxidase assembly factor COX19
CP	ceruloplasmin
CUTA	cutA divalent cation tolerance homolog
CUTC	cutC copper transporter
DBH	dopamine beta-hydroxylase
DCT	dopachrome tautomerase
F5	coagulation factor V
F8	coagulation factor VIII
FKBP4	FKBP prolyl isomerase 4
GPC1	glypican 1
HAMP	hepcidin antimicrobial peptide
HEPH	hephaestin
HEPHL1	hephaestin like 1
IL1A	interleukin 1 alpha
LACC1	laccase domain containing 1
LOX	lysyl oxidase
LOXL1	lysyl oxidase like 1
LOXL2	lysyl oxidase like 2
LOXL3	lysyl oxidase like 3
LOXL4	lysyl oxidase like 4
MMGT1	membrane magnesium transporter 1

MOXD1	monooxygenase DBH like 1
MOXD2P	monooxygenase DBH like 2, pseudogene
MT1A	metallothionein 1A
MT1B	metallothionein 1B
MT1DP	metallothionein 1D, pseudogene
MT1E	metallothionein 1E
MT1F	metallothionein 1F
MT1G	metallothionein 1G
MT1H	metallothionein 1H
MT1HL1	metallothionein 1H like 1
MT1JP	metallothionein 1J, pseudogene
MT1L	metallothionein 1L, pseudogene
MT1M	metallothionein 1M
MT1X	metallothionein 1X
MT2A	metallothionein 2A
MT3	metallothionein 3
MT4	metallothionein 4
MT-CO1	mitochondrially encoded Cytochrome c oxidase subunit 1
MT-CO2	mitochondrially encoded Cytochrome c oxidase subunit 2
MUC2	Mucin-2
OR5AR1	olfactory receptor family 5 subfamil
P2RX4	purinergic receptor P2X 4
PAM	peptidylglycine alpha-amidating mono
PARK7	Parkinsonism associated deglycase
PRND	prion like protein doppel
PRNP	prion protein
RNF7	ring finger protein 7
S100A12	S100 calcium binding protein A12
S100A13	S100 calcium binding protein A13
S100A5	S100 calcium binding protein A5
SCO1	synthesis of cytochrome C oxidase 1
SCO2	synthesis of cytochrome C oxidase 2
SLC11A2	solute carrier family 11 member 2
SLC31A1	solute carrier family 31 member 1
SLC31A2	solute carrier family 31 member 2
SNAI3	snail family transcriptional repress
SNCA	synuclein alpha
SNCB	synuclein beta
SNCG	synuclein gamma
SOD1	superoxide dismutase 1
SOD3	superoxide dismutase 3
STEAP2	STEAP2 metalloreductase
STEAP3	STEAP3 metalloreductase
STEAP4	STEAP4 metalloreductase

SUMF1	sulfatase modifying factor 1
TP53	tumor protein p53
TYR	tyrosinase

TableS2. Copper binding protein-related deferentially expressed genes and their relationship with OS, and their coefficients in univariate Cox regression

Gene	Coefficient	HR	95% CI	p
ALB	-0.356	0.700	0.456 - 1.074	0.103
ANG	0.291	1.338	1.279 - 1.4	<0.001
AOC1	5.427	227.511	40.043 - 1292.648	<0.001
DBH	0.100	1.105	0.734 - 1.664	0.632
F5	-0.061	0.941	0.909 - 0.973	<0.001
HEPHL1	2.950	19.099	4.159 - 87.714	<0.001
IL1A	0.473	1.604	1.429 - 1.8	<0.001
LOX	0.048	1.049	1.041 - 1.057	<0.001
LOXL1	0.075	1.078	1.064 - 1.091	<0.001
LOXL2	0.022	1.023	1.017 - 1.028	<0.001
MOXD1	0.026	1.027	1.022 - 1.032	<0.001
MT1DP	0.210	1.234	1.174 - 1.297	<0.001
MT1H	0.053	1.054	1.034 - 1.075	<0.001
MUC2	-1.382	0.251	0.009 - 6.717	0.410
PRND	0.055	1.056	0.978 - 1.141	0.160
S100A12	0.277	1.319	1.228 - 1.415	<0.001
S100A5	2.570	13.070	8.61 - 19.841	<0.001
SNCB	-0.009	0.991	0.987 - 0.995	<0.001
SNCG	-0.023	0.977	0.969 - 0.986	<0.001
STEAP3	0.052	1.054	1.047 - 1.061	<0.001

Supplementary Figures： Figure S1, Figure S2, Figure S3

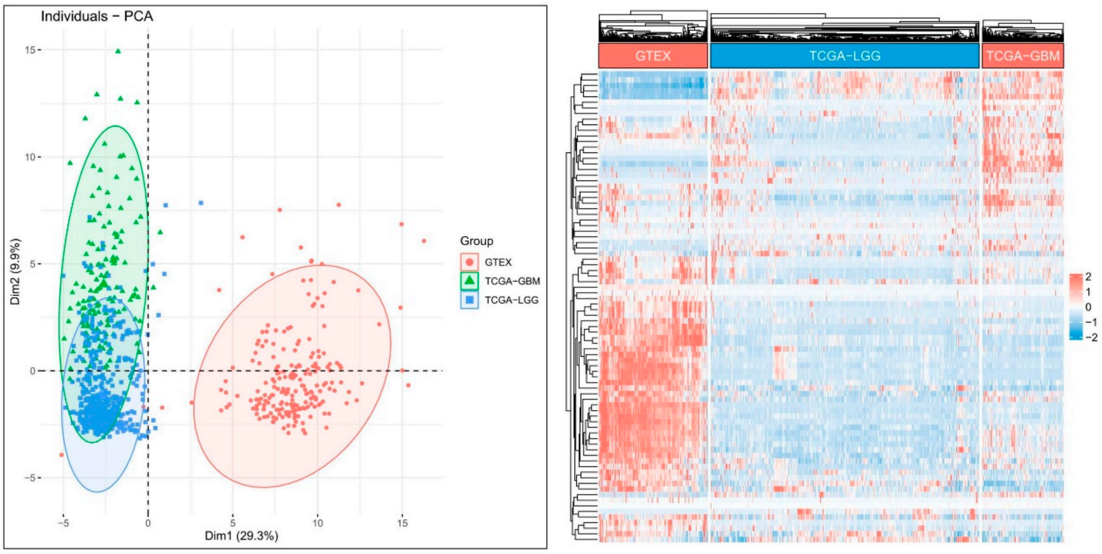

Figure S1. PCA and expression heatmap of Transcriptomic expressions of the copper binding proteins. The transcriptomic expressions of all the copper binding proteins were extracted from the TCGA-GBM, TCGA-LGG and GTEX normal brain cortex datasets, and a total of 78 genes matched. The PCA and heatmap of the gene expressions showed distinct profiles among the three datasets

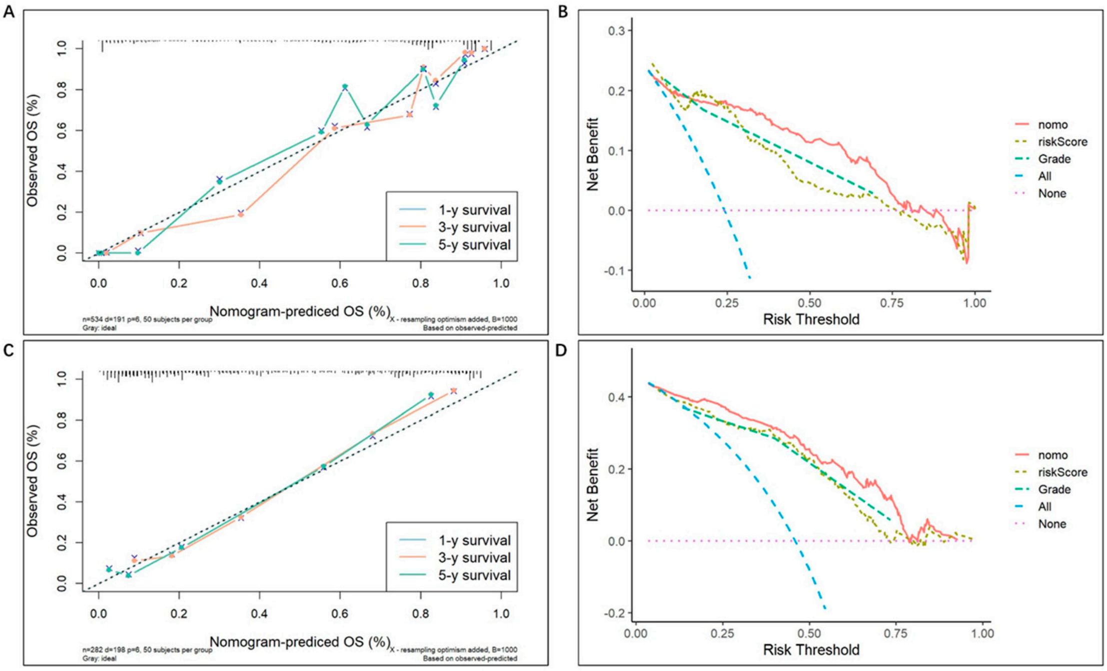

Figure S2. Assessment of the nomogram. Calibration plot of the nomogram for 1- , 3- and 5-year OS based on (A) TCGA and (C) CGGA datasets. DCA of the nomogram in the (B) TCGA and (D) CGGA cohorts.

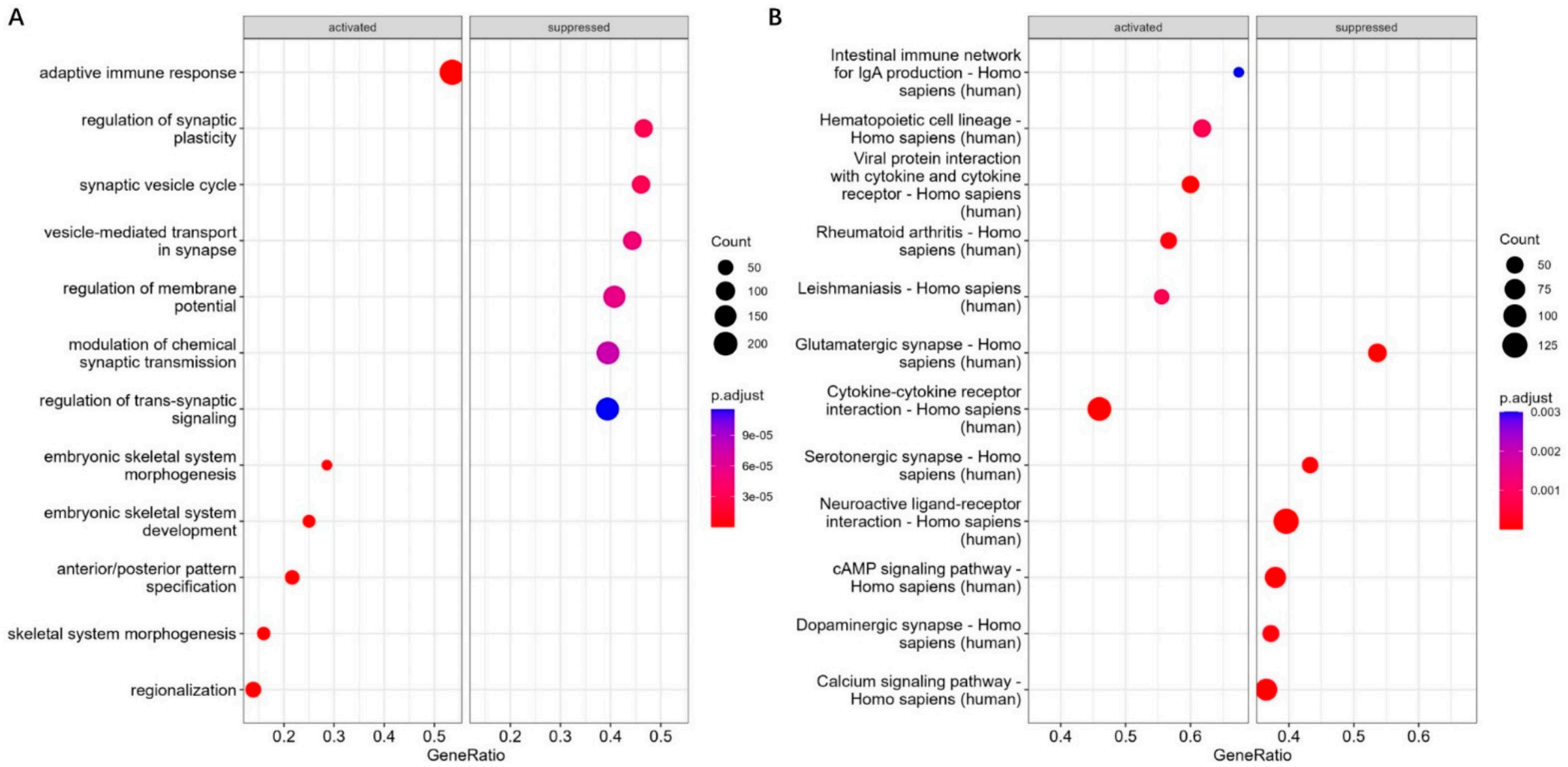

Figure S3. Functional enrichment analysis of the risk model. A, the top 12 biological processes in the high-risk group in the TCGA cohort. B, the top 30 enriched KEGG pathways in the high-risk group.
